# A triple combination of atorvastatin, celecoxib and tipifarnib strongly inhibits pancreatic cancer cells and xenograft pancreatic tumors

**DOI:** 10.3892/ijo.2014.2350

**Published:** 2014-03-19

**Authors:** NING DING, XIAO-XING CUI, ZHI GAO, HUARONG HUANG, XINGCHUAN WEI, ZHIYUN DU, YONG LIN, WEICHUNG JOE SHIH, ARNOLD B. RABSON, ALLAN H. CONNEY, CHUNHONG HU, XI ZHENG

**Affiliations:** 1 Second Xiangya Hospital, Central South University, Changsha 410011, P.R. China;; 2 Susan Lehman Cullman Laboratory for Cancer Research, Department of Chemical Biology, Ernest Mario School of Pharmacy, Rutgers, The State University of New Jersey, Piscataway, NJ 08854, USA;; 3 Allan H. Conney Laboratory for Anticancer Research, Guangdong University of Technology, Guangzhou 510006, P.R. China;; 4 Division of Biometrics, School of Public Health, University of Medicine and Dentistry of New Jersey;; 5 Rutgers Cancer Institute of New Jersey, New Brunswick, NJ 08903, USA

**Keywords:** statin, cyclooxygenase-2, farnesyl transferase, Ras, pancreatic cancer

## Abstract

Because K-Ras mutation and cyclooxygenase-2 (COX-2) overexpression are hallmarks of majority of pancreatic cancer patients, an approach to inhibit the progression and growth of pancreatic cancer using the simultaneous administration of agents that inhibit the function of both targets, should be considered. In the present study, we assessed the effects of atorvastatin (Lipitor), celecoxib (Celebrex) and tipifarnib (Zarnestra) on the growth of human pancreatic cancer. In the *in vitro* studies, we found that treatment of human pancreatic tumor cells with a combination of atorvastatin, celecoxib and tipifarnib had a stronger inhibitory effect on growth and a stronger stimulatory effect on apoptosis than each drug alone or for any combination of two drugs. We also found that treatment of Panc-1 cells with a combination of all three drugs strongly decreased the levels of phosphorylated Erk1/2 and Akt. In an animal model of xenograft tumors in severe combined immunodeficient (SCID) mice, we found that daily i.p. injections of a combination of atorvastatin, celecoxib and tipifarnib had a stronger inhibitory effect on the growth of the tumors in mice than each drug alone or for any combination of two drugs. The results of our study indicate that a combination of atorvastatin, celecoxib and tipifarnib may be an effective strategy for the treatment of pancreatic cancer.

## Introduction

**Figure 1.**
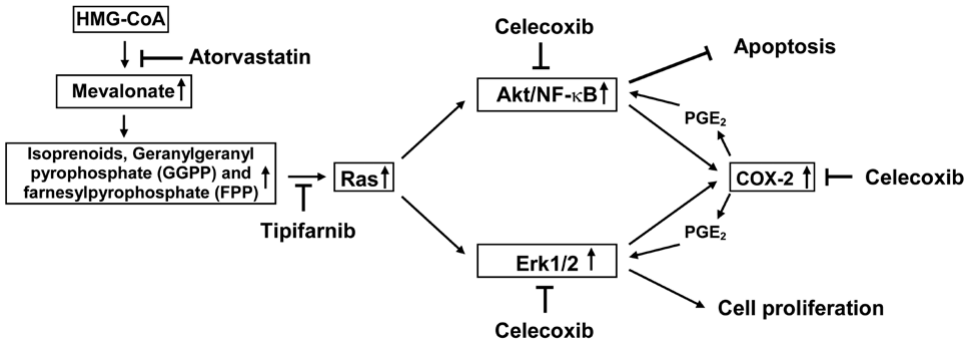
Proposed inhibitory effects of atorvastatin, celecoxib and tipifarnib.

Pancreatic cancer is the 4th leading cause of cancer deaths in the United States (1). Conventional therapies such as surgery, radiation, chemotherapy or a combination of these fail to substantially alter the course of pancreatic cancer, and the prognosis for these patients remains extremely poor with a 5-year survival of only 5% (1) and a median survival of <6 months that has remained unchanged for the last three decades (2,3). The long-term goal of our research is to develop an effective strategy for inhibiting the progression and growth of pancreatic cancer. Ras mutation and overexpression of cyclooxygenase-2 (COX-2) are present in the majority of pancreatic cancer patients (2–5). Ras mutation results in constitutive activation of Erk1/2 and PI3K/Akt pathways leading to increased cell proliferation and decreased apoptosis in pancreatic cancer cells (6). Overexpression of COX-2 results in increased production of prostaglandins and also the activation of Erk1/2 and Akt (7,8). An approach for inhibiting the progression and growth of pancreatic cancer is the simultaneous use of agents that inhibit the function of both Ras and COX-2. A combination of these agents may synergize to suppress pancreatic cancer growth and stimulate apoptosis. We hypothesize that simultaneous inhibition of HMG-CoA reductase with atorvastatin (Lipitor), inhibition of farnesyl transferase with tipifarnib (Zarnestra) and inhibition of COX-2 with celecoxib (Celebrex) will synergistically inhibit downstream readouts of activated ras and elevated COX-2 (activated Akt/NFκB and activated Erk1/2) thereby inhibiting proliferation and stimulating apoptosis. This hypothesis is illustrated in Fig. 1. The drugs chosen for this study are relatively non-toxic, work by different mechanisms and can readily be utilized for a clinical trial. Atorvastatin and celecoxib are in broad clinical use and tipifarnib is a farnesyl transferase inhibitor that has been extensively tested in clinical trials (9–12). To the best of our knowledge the simultaneous targeting of Ras and COX-2 pathways in pancreatic cancer growth is novel.

To test our hypothesis that simultaneous inhibition of the Ras and COX-2 pathways will potently inhibit the growth and induce apoptosis in pancreatic cancer cells *in vitro* and *in vivo*, we investigated the effects of atorvastatin, celecoxib and tipifarnib alone or in combination on the growth and apoptosis of human pancreatic cancer Panc-1 cells cultured *in vitro* or grown as xenograft tumors in SCID mice. We found that treatment of Panc-1 cells with a combination of atorvastatin, celecoxib and tipifarnib had a stronger inhibitory effect on proliferation and a stronger stimulatory effect on apoptosis than any of the drugs alone or for any combination of two drugs. We also found that treatment of tumor-bearing SCID mice with a combination of atorvastatin, celecoxib and tipifarnib had a stronger inhibitory effect on the growth of Panc-1 tumors in these mice than for any of the three drugs alone or for any combination of two drugs.

## Materials and methods

### Cell culture and reagents

Panc-1 cells were obtained from Dr Pamela Crowell (Indiana University - Purdue University Indianapolis, Indianapolis, IN). Luciferase-expressing Panc-1 cells were obtained from Dr Bharart Aggarwal (The University of Texas M.D. Anderson Cancer Center, Houston, TX). Atorvastatin and celecoxib were provided by the National Cancer Institute’s Repository. Tipifarnib was provided by Johnson & Johnson Pharmaceutical Research and Development (Raritan, NJ). Propylene glycol, polysorbate 80, benzyl alcohol, ethanol and DMSO were purchased from Sigma (St. Louis, MO). Matrigel was obtained from BD Biosciences (Bedford, MA). Dulbecco’s modified Eagle’s medium (DMEM) tissue culture medium, penicillin-streptomycin, L-glutamine and fetal bovine serum (FBS) were from Gibco (Grand Island, NY). Panc-1 cells were maintained in DMEM culture medium containing 10% FBS that was supplemented with penicillin (100 U/ml)-streptomycin (100 *μ*g/ml) and L-glutamine (300 *μ*g/ml). Cultured cells were grown at 37°C in a humidified atmosphere of 5% CO_2_ and were passaged twice a week. Panc-1 cells were initially seeded at a density of 0.2×10^5^ cells/ml in 35-mm tissue culture dishes (2 ml/dish) for assays of proliferation and apoptosis, and seeded at a density of 1×10^5^ cells/ml of medium in 100 mm culture dishes (10 ml/dish) for the western blot analysis. Atorvastatin, celecoxib and tipifarnib were dissolved in DMSO and the final concentration of DMSO in all experiments was 0.2%.

### Determination of the number of viable cells

The number of viable cells after each treatment was determined using a hemacytometer under a light microscope (Nikon Optiphot, Nikon, Tokyo, Japan). Cell viability was determined by the trypan blue exclusion assay, which was done by mixing 80 *μ*l of cell suspension and 20 *μ*l of 0.4% trypan blue solution for 2 min. Blue cells were counted as dead cells and the cells that did not absorb dye were counted as live cells.

### Morphological assessment of apoptotic cells

Apoptosis was determined by morphological assessment in cells stained with propidium iodide (13). Briefly, cytospin slides were prepared after each experiment and cells were fixed with acetone/methanol (1:1) for 10 min at room temperature, followed by 10 min with propidium iodide staining (1 *μ*g/ml in PBS) and analyzed using a fluorescence microscope (Nikon Eclipse TE200, Nikon). Apoptotic cells were identified by classical morphological features including nuclear condensation, cell shrinkage, and formation of apoptotic bodies (13). At least 200 cells were counted in each sample and the percentage of apoptotic cells is presented.

### Western blot analysis

After treatment, Panc-1 cells were washed with ice-cold PBS and lysed with 800 *μ*l of lysis buffer (10 mM Tris-HCl, pH 7.4, 50 mM sodium chloride, 30 mM sodium pyrophosphate, 50 mM sodium fluoride, 100 *μ*M sodium orthovandate, 2 mM iodoacetic acid, 5 mM ZnCl_2_, 1 mM phenylmethylsulfonyl fluoride and 0.5% Triton X-100). The homogenates were centrifuged at 12,000 x g for 15 min at 4°C. The protein concentration of whole cell lysates was determined with a Bio-Rad protein assay kit (Bio-Rad, Hercules, CA). Equal amounts (20 *μ*g) of protein were then resolved on a 10% Criterion Precast Gel (Bio-Rad) and transferred to a PVDF membrane. The membrane was then probed with anti-phosphorylated Erk1/2 and Akt primary antibodies (Cell Signaling Technology, Beverly, MA). After hybridization with primary antibody, the membrane was washed with Tris-buffered saline three times, then incubated with secondary antibodies conjugated with infrared-dye (Cell Signaling Technology) and washed with Tris-buffered saline three times. Labeled proteins were visualized using the Odyssey infrared imaging system (LI-COR Biosciences, Lincoln, NE). The extent of protein loading was determined by blotting for β-actin.

### Subcutaneous and orthotopic xenograft Panc-1 tumors in immunodeficient mice

Female severe combined immunodeficient (SCID) mice (6–7 weeks old) were obtained from Taconic Farms Inc (Germantown, NY). The animals were housed in sterile filter-capped microisolator cages and provided with sterilized food and water. For subcutaneous xenograft tumors, pancreatic cancer Panc-1 cells (2×10^6^ cells/0.1 ml/mouse) suspended in 50% Matrigel (Collaborative Research, Bedford, MA) in DMEM medium were injected subcutaneously into the right flank of the mice. After 4–6 weeks, mice with Panc-1 tumors (0.6–1.0 cm wide and 0.6–1.0 cm long) were injected with vehicle (5 *μ*l/g body weight), atorvastatin (2 *μ*g/g), celecoxib (2 *μ*g/g), tipifarnib (0.8 *μ*g/g), atorvastatin (2 *μ*g/g) + celecoxib (2 *μ*g/g), atorvastatin (2 *μ*g/g) + tipifarnib (0.8 *μ*g/g), celecoxib (2 *μ*g/g) + tipifarnib (0.8 *μ*g/g) or atorvastatin (2 *μ*g/g) + celecoxib (2 *μ*g/g) + tipifarnib (0.8 *μ*g/g) once a day for 30 days. In all experiments, animals in the different experimental groups received the same amount of vehicle (5 *μ*l/g body weight) which consisted of propylene glycol, polysorbate 80, benzyl alcohol, ethanol and water (40:0.5:1:10:48.5) (14). Tumor size (length x width) and body weight were measured every third day. For the orthotopic xenograft experiment, luciferase-expressing Panc-1 cells (15) harvested from subconfluent cultures were injected into the pancreas of female SCID mice. In this procedure, mice were anesthetized with ketamine-xylazine solution, a small left abdominal flank incision was made, and Panc-1 cells (1×10^6^) in 100 *μ*l DMEM medium were injected into the subcapsular region of the pancreas using a 27-gauge needle. To prevent leakage, a cotton swab was held for 1 min over the site of injection. The abdominal wound was closed with wound clips (Braintree Scientific, Inc., Braintree, MA). Three weeks after injection of tumor cells, the mice were randomized into two groups based on the IVIS imaging. The control group received i.p. injections with vehicle (5 *μ*l/g body weight) and the combination treatment group received i.p. injections with atorvastatin (2 *μ*g/g) + celecoxib (2 *μ*g/g) + tipifarnib (0.8 *μ*g/g) once a day for 28 days. Each group had 4 mice. For the IVIS imaging, the mice were anesthetized with isoflurane and injected i.p. with luciferin. The mice were then placed into the imaging chamber of the IVIS imaging system (Xenogen Corporation, San Diego, CA) and a bioluminescence image was taken. All animal experiments were carried out under an Institutional Animal Care and Use Committee (IACUC)-approved protocol.

### Statistical analyses

The analyses of percentage of initial tumor size were based on a repeated measurement model (16). The treatment effects were assessed by comparing the rates of change over time between treatment groups (i.e. comparing the slopes and/or quadratic trends between treatment groups). Heterogeneous autoregressive correlation structure was used to account for the within-mice correlation. The analysis of variance (ANOVA) model with Tukey-Kramer adjustment (17) was used for the comparison of tumor size and body weight among different treatment groups at the end of the treatment.

## Results

### Effects of atorvastatin, celecoxib and tipifarnib on the growth and apoptosis of cultured Panc-1 cells

**Figure 2.**
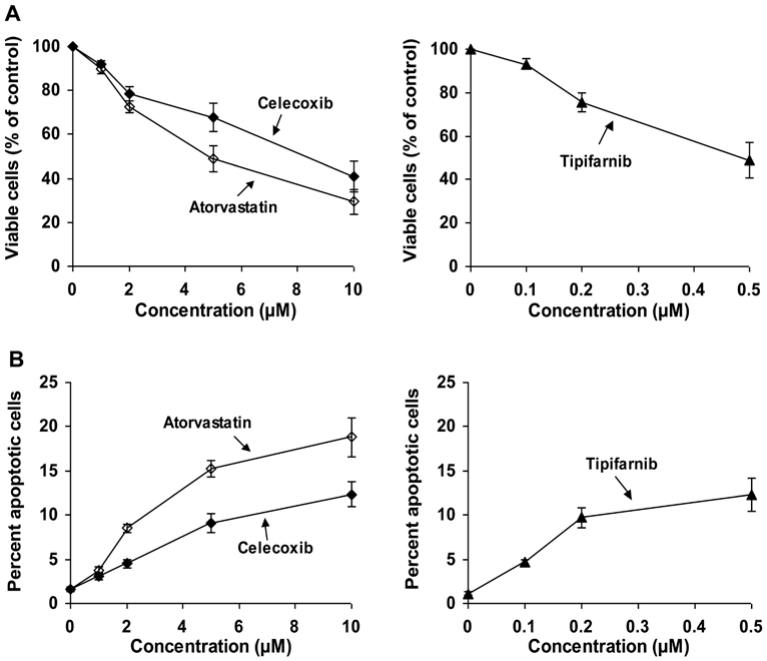
Effects of atorvastatin, celecoxib or tipifarnib on proliferation and apoptosis of Panc-1 cells. Panc-1 cells were seeded at a density of 0.2×10^5^ cells/ml in 35-mm tissue culture dishes (2 ml/dish) and incubated for 24 h. The cells were then treated with DMSO (2 *μ*l/ml) or with various concentrations of atorvastatin (1–10 *μ*M), celecoxib (1–10 *μ*M) or tipifarnib (0.1–0.5 *μ*M) in DMSO for 96 h. Each incubation mixture contained the same amount of solvent (DMSO; 2 *μ*l/ml). (A) The number of viable cells was measured by a trypan blue exclusion assay and expressed as a percentage of solvent-treated control. (B) The number of apoptotic cells was determined by morphological assessment. Each value is the mean ± SE from three separate experiments.

Dose-response studies on the effects of atorvastatin, celecoxib or tipifarnib on the growth and apoptosis of cultured Panc-1 cells using our previously utilized methodology (13,14,18) are shown in Fig. 2. The concentration of atorvastatin, celecoxib or tipifarnib alone to achieve 50% inhibition of the growth of Panc-1 cells was 5, 8 and 0.5 *μ*M, respectively (Fig. 2A) whereas a combination of the three drugs at 1, 1 and 0.1 *μ*M, respectively, also inhibited Panc-1 growth by ∼50% (Table I). Treatment of Panc-1 cells with a combination of the 3 drugs had a stronger inhibitory effect on proliferation and a stronger stimulatory effect on apoptosis than the individual drugs alone or for any combination of two drugs (Table I).

### Effects of atorvastatin, celecoxib and tipifarnib on the levels of activated Akt and Erk1/2 in cultured Panc-1 cells

**Figure 3.**
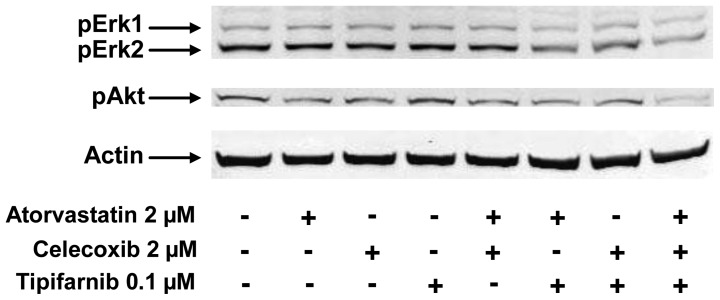
Effects of atorvastatin, celecoxib or tipifarnib alone or in combination on phosphorylation of Erk1/2 and Akt. Panc-1 cells were seeded at a density of 0.1×10^6^ cells/ml of medium in 100-mm tissue culture dishes and incubated for 24 h. The cells were then treated with atorvastatin (2 *μ*M), celecoxib (2 *μ*M) or tipifarnib (0.1 *μ*M) alone or in combination for 24 h. Western blot analysis with anti-phosphorylated Erk1/2 (#4376, Cell Signaling Technology) and anti-phosphorylated Akt (#9275, Cell Signaling Technology) was used to determine the expression of phosphorylated-Erk and phosphorylated-Akt.

The levels of activated Akt and Erk1/2 in Panc-1 cells were evaluated by western blot analysis using anti-phospho-Akt (pAkt) and anti-phospho-Erk1/2 (pErk1/2) antibodies (both from Cell Signaling Technology). In these experiments, Panc-1 cells were treated with atorvastatin (2 *μ*M), celecoxib (2 *μ*M) or tipifarnib (0.1 *μ*M) alone or in combination for 24 h and analyzed by western blot analysis. As shown in Fig. 3, treatment of Panc-1 cells with each drug alone or any combination of two drugs for 24 h had little or no effect on the level of pAkt in the cells, but the combination of atorvastatin, celecoxib and tipifarnib caused a strong decrease in the level of pAkt (Fig. 3). Treatment of Panc-1 cells with each drug alone or with a combination of atorvastatin and celecoxib had little or no effect on the level of pErk1/2 in the cells (Fig. 3). Treatment of Panc-1 cells with atorvastatin + tipifarnib or celecoxib + tipifarnib resulted in modestly decreased pErk1/2 in the cells while a combination of atorvastatin, celecoxib and tipifarnib caused a much stronger decrease in pErk1/2 (Fig. 3).

### Effects of i.p. injections of atorvastatin, celecoxib and tipifarnib alone or in combination on the growth of Panc-1 xenograft tumors in immunodeficient mice

**Figure 4.**
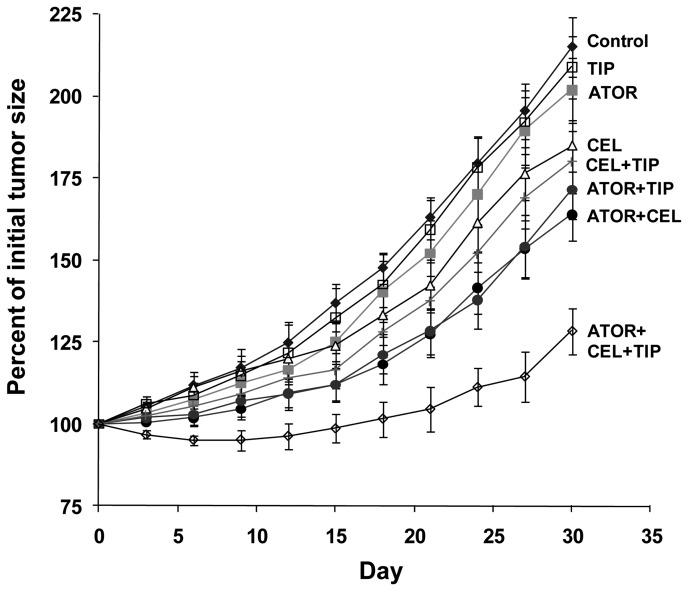
Inhibitory effect of i.p. injection of atorvastatin, celecoxib or tipifarnib alone or in combination on the growth of subcutaneous Panc-1 tumors in SCID mice. Female SCID mice were injected subcutaneously with Panc-1 cells (2×10^6^ cells/0.1 ml) suspended in 50% Matrigel and RPMI medium. Injections of drug were started in mice after they had a tumor (0.6–1.0 cm wide and 0.6–1.0 cm long). The mice (6/group) received daily i.p. injections of vehicle (see below) or atorvastatin (ATOR; 2 *μ*g/g body weight), celecoxib (CEL; 2 *μ*g/g) or tipifarnib (TIP; 0.8 *μ*g/g) alone or in combination. Each value is the mean ± SE. The linear trend in tumor growth for the atorvastatin + celecoxib + tipifarnib group was significantly different from that for any other group (p=0.0034).

Female SCID mice with subcutaneous Panc-1 tumors measuring 0.5–1.0 cm in length and 0.5–1.0 cm in width were injected i.p. with celecoxib (2 *μ*g/g body weight/day), atorvastatin (2 *μ*g/g body weight/day) or tipifarnib (0.8 *μ*g/g body weight/day) alone or in combination. The combination of celecoxib, atorvastatin and tipifarnib had a strong inhibitory effect on tumor growth, whereas injection of tipifarnib or atorvastatin alone was inactive and celecoxib alone or celecoxib plus tipifarnib had only a small inhibitory effect on tumor growth, and administration of atorvastatin plus celecoxib or atorvastatin plus tipifarnib had a moderate inhibitory effect on tumor growth (Fig. 4). Quadratic trends of tumor growth were similar for all groups. The linear trends in tumor growth for all the treatment groups were significantly different from the vehicle control group (p≤0.0021) except for the atorvastatin group and tipifarnib group. The linear trends for the atorvastatin group and tipifarnib group were not significantly different from the control (p-values were 0.1294 and 0.4996, respectively). The linear trend in tumor growth for the atorvastatin + celecoxib + tipifarnib group was significantly different from that for any other group (p≤0.0034). The strong inhibitory effect of the administration of a combination of atorvastatin, celecoxib and tipifarnib on tumor growth lasted for ∼4 weeks followed by a partial restoration of tumor growth (Fig. 4). At the end of the study when the animals were sacrificed, the mean ± SE for the percent of initial body weight was 92.2±2.0% for the vehicle treated control group, 92.9±2.3% for the atorvastatin group, 87.8±2.5% for the celecoxib group, 90.7±1.3% for the tipifarnib group, 90.0±2.1% for the atorvastatin + celecoxib group, 86.3±2.7% for the atorvastatin + tipifarnib group, 90.9±1.4% for the celecoxib + tipifarnib group and 89.3±1.9% for the atorvastatin + celecoxib + tipifarnib group. Statistical analysis using ANOVA with the Tukey-Kramer multiple comparison test showed that the difference in the percent of initial body weight between the control group and any of the treatment groups was not statistically significant (p>0.05).

**Figure 5.**
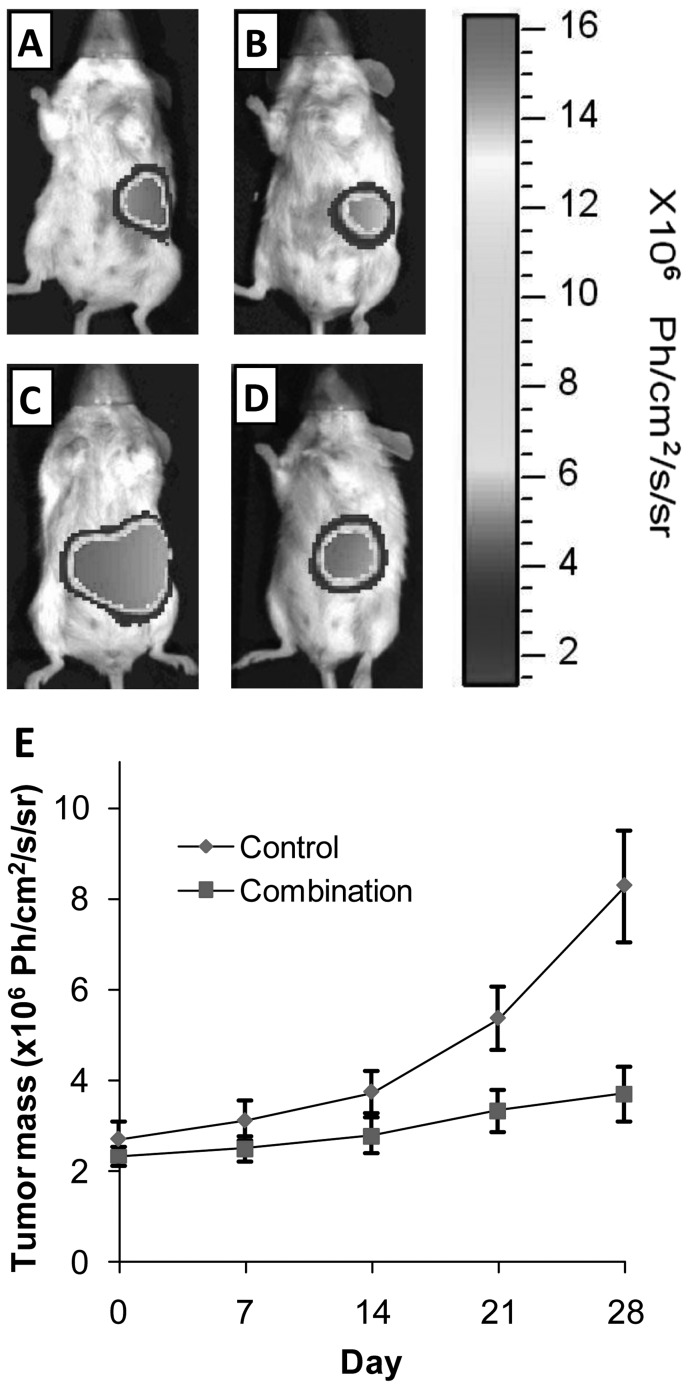
Inhibitory effect of i.p. injections of atorvastatin, celecoxib and tipifarnib in combination on the growth of orthotopic Panc-1 tumors in SCID mice. Female SCID mice were injected orthotopically with Panc-1 luc cells (1×10^6^ cells/mouse). After 2–3 weeks, mice with established Panc-1 tumors were randomized into two groups. One group of mice received daily i.p. injections with solvent and the other group of mice received daily i.p. injections with atorvastatin (2 *μ*g/g) + celecoxib (2 *μ*g/g) + tipifarnib (0.8 *μ*g/g). Tumor size of each mouse was determined once a week using the IVIS system. Representative images are shown. (A and B) Mice before treatment. (C) An image taken from a mouse in the solvent control group 28 days after treatment. (D) An image taken from a mouse in the combination treatment group 28 days after treatment. (E) Each value for tumor mass is the mean ± SE from 4 mice.

In an additional experiment, we determined the effect of atorvastatin, celecoxib and tipifarnib in combination on the growth of orthotopic Panc-1 xenograft tumors in SCID mice. As shown in Fig. 5, treatment of the mice with daily i.p. injections of atorvastatin (2 *μ*g/g body weight/day) + celecoxib (2 *μ*g/g body weight/day) + tipifarnib (0.8 *μ*g/g body weight/day) strongly inhibited the growth of orthotopic Panc-1 xenograft tumors for the duration of the 28-day study. Statistical analysis using the Student’s t-test showed that the difference in tumor size between the control group and the combination treatment group at the end of the study was statistically significant (p<0.01). The difference in the percent of initial body weight between the control group and the combination treatment group was not statistically significant (Student’s t-test; p>0.05).

## Discussion

In the present study, we showed that daily i.p. injections of SCID mice with atorvastatin, celecoxib and tipifarnib in combination had a stronger inhibitory effect on the growth of Panc-1 xenograft tumors than atorvastatin, celecoxib or tipifarnib used alone or for any combination of two drugs (Fig. 4). Although the triple drug combination had a strong inhibitory effect on tumor growth, there was no effect on body weight. We also found that atorvastatin, celecoxib and tipifarnib in combination inhibited the growth, induced apoptosis and decreased phosphorylation of Akt and Erk1/2 in cultured Panc-1 cells (Figs. 2 and 3). To the best of our knowledge, this is the first report on the combined inhibitory effect of atorvastatin, celecoxib and tipifarnib on the growth of pancreatic cancer cells cultured *in vitro* or grown as xenograft tumors in immunodeficient mice.

Tipifarnib is a selective non-peptidomimetic inhibitor of farnesyltransferase. Preclinical studies demonstrated that tipifarnib competitively inhibits farnesylation of K-ras peptides at nanomolar concentrations. Tipifarnib also inhibits proliferation of pancreatic cancer cells cultured *in vitro* and grown as xenograft tumors in immunodeficient mice (19). However, the results of clinical studies showed that tipifarnib did not exhibit single-agent antitumor activity in patients with previously untreated metastatic pancreatic cancer (12,20,21). A possible explanation for these negative results of clinical trials is that Ras can be geranylgeranylated, an alternative lipidation that can substitute for farnesylation (22), and this reaction may not be inhibited by farnesyltransferase inhibitors. Therefore, agents such as 3-hydroxy-3-methylglutaryl-CoA (HMG-CoA) reductase inhibitors that can reduce both geranylgeranyl pyrophosphate (GGPP) and farnesylpyrophosphate (FPP) may enhance the effectiveness of tipifarnib. Atorvastatin is a member of the statin family of drugs that inhibit HMG-CoA reductase, a rate limiting enzyme in cholesterol biosynthesis, and this drug is used clinically as a safe and effective agent for the control of hypercholesterolemia (23). HMG-CoA reductase inhibition leads to reduced synthesis of isoprenoids, GGPP and FPP (24,25). In the present study, we found that a combination of atorvastatin and tipifarnib decreased the level of pErk1/2 while atorvastatin or tipifarnib alone had little or no effect on the level of pErk1/2. Although further studies are needed to determine the inhibitory effect of atorvastatin and tipifarnib on activation of Ras protein, our results indicated that a combination of these two drugs more potently inhibited the Ras down-stream effector Erk1/2 than either drug alone.

Celecoxib, a selective COX-2 inhibitor, has been shown previously to inhibit the growth of human pancreatic cancer cell lines (26,27). Recent studies showed that celecoxib inhibited angiogenesis, tumor growth and metastasis in pancreatic xenograft tumors (28,29), and celecoxib also inhibited pancreas cancer formation in a COX-2 overexpressing mouse (30). Although treatment with celecoxib alone did not influence tumor growth in tumor-bearing mice (30), the possibility that a combination of celecoxib, atorvastatin and tipifarnib will inhibit pancreas tumor growth in this genetic model of pancreas cancer has not yet been explored. The present study provides evidence that the combination of celecoxib, atorvastatin and tipifarnib have a strong inhibitory effect on the growth of both subcutaneous and orthotopic Panc-1 xenograft tumors in SCID mice.

Although loss of effectiveness of the triple drug combination after 4 weeks of inhibition of tumor growth is suggested by the last data point in Fig. 4, no loss of effectiveness was observed during a 4 week treatment interval in a second study with an orthotopic pancreas tumor model (Fig. 5). Additional studies are needed to strengthen data on a possible loss of effectiveness of the triple drug combination on tumor growth with continued therapy beyond 4 weeks. The possible loss of effectiveness of drug treatment after 4 weeks of therapy could be because chronic drug treatment (or vehicle) stimulated metabolic inactivation of the drugs (enzyme induction) or because the tumor cells became intrinsically resistant to drug administration. In a separate study in male SCID mice, feeding a combination of celecoxib (0.05% in diet) and atorvastatin (0.02% in diet) for 2 weeks resulted in substantially lower serum levels of atorvastatin and its metabolites than in the serum of mice fed atorvastatin alone (unpublished observations). These results suggest that increasing the dose of atorvastatin after 2–3 weeks of treatment may prevent the loss of effectiveness at late time intervals. Additional studies are needed to determine whether treating the mice with atorvastatin, celecoxib and tipifarnib stimulates the hepatic metabolism of these drugs to inactive products or whether tumor cells become intrinsically resistant to drug treatment. The strong inhibitory effect of a combination of atorvastatin, celecoxib and tipifarnib (relatively non-toxic drugs) on the growth of pancreas tumors provide a rationale for a clinical trial to determine the effectiveness of the simultaneous administration of atorvastatin, celecoxib and tipifarnib possibly in combination with gemcitabine on the growth of pancreas tumors in pancreas cancer patients or to determine whether the triple drug combination will prevent the recurrence of pancreas cancer in patients who have undergone surgery by the Whipple procedure to remove their pancreas cancer. It is well-known that patients who have had surgical removal of their pancreas tumor by the Whipple procedure have a high risk for recurrence of pancreas cancer (31,32).

## Figures and Tables

**Table I.** Effects of atorvastatin, celecoxib and tipifarnib alone or in combination on the growth and apoptosis of Panc-1 cells.

Treatment	Viable cells (% of control)	Apoptosis (% of cells)
Control	100	1.5±0.5
Atorvastatin (1 *μ*M)	91.1±2.3	4.3±0.5
Celecoxib (1 *μ*M)	93.4±1.6	3.8±0.6
Tipifarnib (0.1 *μ*M)	90.3±1.1	5.6±0.4
Atorvastatin (1 *μ*M) + celecoxib (1 *μ*M)	79.7±6.9	8.8±1.1
Atorvastatin (1 *μ*M) + tipifarnib (0.1 *μ*M)	80.0±2.3	6.7±0.2
Celecoxib (1 *μ*M) + tipifarnib (0.1 *μ*M)	83.7±2.8	6.3±0.8
Atorvastatin (1 *μ*M) + celecoxib (1 *μ*M) + tipifarnib (0.1 *μ*M)	49.9±5.2	20.5±3.1

Panc-1 cells were seeded at a density of 2×10^4^ cells/ml of medium and incubated for 24 h. The cells were then treated with atorvastatin, celecoxib or tipifarnib for 96 h. DMSO was used as the vehicle for each drug and the final concentration of DMSO for each incubation was 0.2%. The number of viable cells was counted by using the trypan blue exclusion assay. Apoptotic cells were determined by morphological assessment. Each value represents the mean ± SE from multiple samples in a single experiment. Statistical analysis using ANOVA with the Tukey-Kramer multiple comparison test showed that the number of viable cells was significantly lower in the atorvastatin + celecoxib + tipifarnib group than in the control or any other treatment groups (p<0.001), and that the percent apoptotic cells was significantly higher in the atorvastatin + celecoxib + tipifarnib group than in the control or any other treatment group (p<0.001).
